# Physical and Oxidative Stabilization of Oil-In-Water Emulsions by Roasted Coffee Fractions: Interface- and Continuous Phase-Related Effects

**DOI:** 10.1021/acs.jafc.2c07365

**Published:** 2023-03-09

**Authors:** Jilu Feng, Karin Schroën, Sylvain Guyot, Agnès Gacel, Vincenzo Fogliano, Claire C. Berton-Carabin

**Affiliations:** † Food Quality and Design Group, Wageningen University and Research, 6708WG Wageningen, Netherlands; ‡ Food Process and Engineering Group, Wageningen University and Research, 6708WG Wageningen, Netherlands; § INRAE, UR BIA, F-44316 Nantes, France; ∥ INRA UR1268 BIA, F-35653 Le Rheu, France

**Keywords:** coffee, emulsions, interface, continuous phase, lipid oxidation, melanoidins

## Abstract

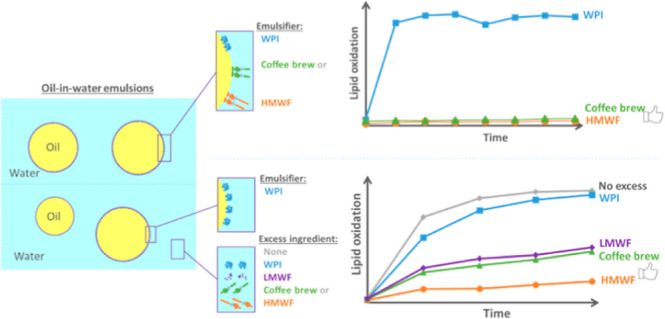

Emulsions fortified with polyunsaturated fatty acids are highly relevant from a nutritional perspective; however, such products are prone to lipid oxidation. In the current work, this is mitigated by the use of natural antioxidants occurring in coffee. Coffee fractions with different molecular weights were extracted from roasted coffee beans. These components were positioned either at the interface or in the continuous phase of emulsions where they contributed to emulsion stability via different pathways. Coffee brew as a whole, and its high-molecular-weight fraction (HMWF), was able to form emulsions with good physical stability and excellent oxidative stability. When added post-homogenization to the continuous phase of dairy protein-stabilized emulsions, all coffee fractions were able to slow down lipid oxidation considerably without altering the physical stability of emulsions, though HMWF was more effective in retarding lipid oxidation than whole coffee brew or low-molecular-weight fraction. This is caused by various effects, such as the antioxidant properties of coffee extracts, the partitioning of components in the emulsions, and the nature of the phenolic compounds. Our research shows that coffee extracts can be used effectively as multifunctional stabilizers in dispersed systems leading to emulsion products with high chemical and physical stability.

## 1 Introduction

It is nowadays well-recognized that higher amounts of ω-3 polyunsaturated fatty acids should be targeted to contribute to healthier diets.^1^ As a result, the food industry strives for developing ω-3-rich products, but that is far from trivial. The presence of several double bonds makes ω-3 polyunsaturated fatty acids vulnerable to oxidation, which results in quality deterioration in foods (e.g., undesirable changes in flavor, nutritional quality, and shelf life).^2,3^ A strategy to counteract lipid oxidation is through the addition of antioxidants, and ideally, these should be natural antioxidants that are preferred by consumers.^4^ Synthetic antioxidants, such as butylated hydroxytoluene and butylated hydroxyanisole (BHA), are known to be highly effective. Alternatively, natural antioxidants (e.g., rosemary extracts and tocopherols) have been used, but the search for natural alternatives is still very much on.

Coffee is a rich source of compounds with potent antioxidant activity, which is modulated by the coffee bean roasting process.^5^ During roasting, on the one hand, natural phenolic compounds (predominantly chlorogenic acids, CGAs) present in the green coffee beans undergo chemical reactions such as isomerization, degradation, and/or oxidation, leading to a reduction of their antioxidant activity,^6^ whereas on the other hand, additional antioxidant activity may be created through the formation of certain Maillard reaction products (MRPs). In particular, melanoidins that are generated during roasting of coffee beans at high temperatures and low water activity^7,8^ could be of interest due to their antioxidant potential. Some MRPs (such as acrylamide, heterocyclic amines, and 5-hydroxymethylfurfural) are potentially toxic and their presence in foods should be kept as low as reasonably achievable,^9^ yet this concern does not apply to melanoidins: they are not bioavailable and have positive functions within the gastrointestinal tract, similar to dietary fibers.^10^ In addition, some volatile heterocyclic compounds (furans, pyrroles, and maltol) formed during roasting have also been reported as potential antioxidants.^11^ The antioxidant mechanisms of coffee components were reported to be mainly related to their ability to break the radical chain reaction cascade by hydrogen donation and to chelate metal ions.^12−14^

Coffee fractions may contribute to the overall antioxidant activity of coffee in various ways. The high antioxidant activity of the high-molecular-weight fraction (HMWF) from coffee brew was attributed to melanoidins to which low-molecular-weight compounds (e.g., phenolic compounds) were bound.^12,15,16^ The low-molecular-weight fraction (LMWF) from coffee brew is in itself rich in phenolic compounds that constitute 70% of the overall antioxidant capacity,^17^ and the remaining effects are expected to be caused by volatile heterocyclic compounds.^18^ Although the antioxidant activity (e.g., metal chelating and radical scavenging activities) of coffee fractions has been widely reported, their ability to inhibit lipid oxidation in food systems (e.g., emulsions) has not been investigated. Unfortunately, the antioxidant activity is not always a good predictor of their efficacy as antioxidants in foods.^19^ This could be due to the complexity of food systems where antioxidants can be partitioned at different locations, and therefore their properties (reactivity) may vary depending on the nature of the environments present (e.g., interactions with other components).

In addition to their intrinsic chemical properties, the effect of chemically active components on lipid oxidation in emulsions largely depends on their localization (i.e., in the oil phase, aqueous phase, or at the interface). It is widely admitted that lipid oxidation initiates at the oil–water interface.^20^ Adsorbed emulsifiers with antioxidant potential (interfacial antioxidants) may therefore promote good oxidative stability of emulsified lipids through various mechanisms, such as free radical scavenging, transition metal chelating, and secondary oxidation product binding.^2^ On the other hand, localization away from the interface may make antioxidants less effective in mitigating the previously mentioned effects, but may still contribute through other mechanisms, e.g., binding of metal ions, therewith delaying initiation of lipid oxidation.^2^ For instance, there is a large proportion of emulsifiers remaining in the continuous phase, and thus, the contribution of these non-adsorbed emulsifiers (in particular, proteins) to retard lipid oxidation could be substantial.^20^

We recently reported the ability of the HMWF from coffee brew to physically stabilize oil-in-water (O/W) emulsions, with the polysaccharide-rich fraction predominantly present at the interface.^21^ Considering their emulsifying properties and well-known antioxidant activity, one can assume that certain coffee fractions could act as antioxidant emulsifiers. The current research was therefore aimed to assess the efficiency of different coffee fractions, either present at the interface or in the continuous phase, to stabilize O/W emulsions, with a particular emphasis on their ability to inhibit lipid oxidation. To achieve this, different coffee fractions (whole coffee brew, HMWF, non-defatted HMWF, and LMWF) were extracted from dark roasted arabica coffee beans. Then, they were either used to make rapeseed oil-in-water (O/W) emulsions, or added to the continuous phase of whey protein isolate (WPI)-stabilized emulsions (with minimal excess WPI remaining in the continuous phase). The physical and oxidative stability of these emulsions were monitored during storage at 40 °C.

## 2 Materials and Methods

### 2.1 Materials

Dark roasted arabica coffee beans and rapeseed oil were obtained from a local supermarket (Wageningen, the Netherlands). Rapeseed oil was stripped with alumina powder (Alumina N, Super I, EcoChrome, MP Biomedicals, France) to remove the surface-active impurities and tocopherols.^22^ WPI (88.11 ± 1.15 wt %, N × 6.25) was obtained from Davisco (Lancy, Switzerland). l-Ascorbic acid, 1,1-diphenyl-2-picryl-hydrazyl (DPPH), 3-(2-pyridyl)-5,6-di(2-furyl)-1,2,4-triazine-5′,5″-disulfonic acid disodium salt (ferene), iron(II) sulfate heptahydrate (FeSO_4_·7H_2_O), CGA (#C3878), caffeic acid, *p*-coumaric, cumene hydroperoxide solution (80%), sodium chloride (NaCl), *para*-anisidine, and *n*-hexane were purchased from Sigma-Aldrich (Saint Louis, MO, USA). Glacial acetic acid, hydrochloric acid (37%), 2-propanol, 1-butanol, ethanol, ethylenediaminetetraacetic acid (EDTA), barium chloride dihydrate (BaCl_2_·2H_2_O), ammonium thiocyanate (NH_4_SCN), and sodium hydroxide (NaOH) were obtained from Merck Millipore (Merck, Germany). Acetonitrile and methanol were purchased from Carlo Erba Reagents (Val de Reuil, France). Dichloromethane was obtained from Actu-All Chemicals B.V. (Oss, The Netherlands). Sodium acetate trihydrate was purchased from VMR (Radnor, PA, USA). Ferulic acid was obtained from Extrasynthèse (Genay, France). All solvents were of at least of analytical grade. Ultrapure water was obtained from a Millipore Milli-Q water purification system (Millipore Corporation, Billerica, MA, USA) and used for all experiments.

### 2.2 Preparation of Coffee Brew

Roasted coffee beans were ground using a Spex sample Prep 6870 cryogenic mill (Minneapolis, Minnesota, US) to pass through a 0.425 mm sieve. The ground coffee was defatted using dichloromethane (1:3, w/v) three times. Coffee brew was prepared by adding 100 g of the defatted ground coffee to 1200 mL of water at 80 °C for 20 min, followed by filtering through a filter paper (Whatman 595, Billerica, MA, US). Part of the filtrate was freeze-dried and stored at −20 °C before use, and the other part of the filtrate was used for further isolation (Section 2.3).

### 2.3 Isolation of HMWF, LMWF, and Non-Defatted HMWF from Coffee Brew

An aliquot of the coffee brew obtained above was subjected to ultrafiltration (10 kDa, Amicon stirred cell, Millipore Co., MA, US). The filtrate was collected and is referred to as LMWF. To the retentate, 100 mL of water was added during three washing steps, which thus became the HMWF. The HMWF was lyophilized and stored at −20 °C. To wrap up, HMWF contains all the components that cannot go through the membrane, whereas LMWF contains all the components that can.

Non-defatted HMWF was extracted similarly as HMWF, except for the defatting step with dichloromethane that was not included. Non-defatted HMWF was prepared to investigate how the defatting step and endogenous lipids affected the physical and oxidative stability of the emulsions.

### 2.4 Carbohydrate, Protein, and Phenolic Group Contents

The total sugar content of the different coffee fractions (coffee brew, HMWF, non-defatted HMWF, and LMWF) in aqueous medium was measured using the phenol-sulfuric acid method.^23^ Nitrogen content was determined using the Dumas method (Interscience Flash EA 1112 series, Thermo Scientific, Breda, The Netherlands), and protein content was estimated using a nitrogen to protein factor of 5.5.^24^ Phenolic group content was evaluated with the Folin–Ciocalteu reagent using CGA as the standard.^25^

### 2.5 Analysis of Unbound Phenolic Compounds by Liquid Chromatography Coupled with Diode Array Detection and Mass Spectrometry

Methanol suspensions (for coffee brew and HMWFs) or dilutions (for LMWF) of the coffee fractions were sonicated for 30 min, then diluted 2-fold with acidified water (0.1 v % formic acid), filtrated on 0.45 μm PTFE filters, and finally injected (2 μL) onto the liquid chromatography coupled with diode array detection and mass spectrometry (LC-DAD-MS) system. Separations were performed on a reverse-phase Purospher STAR Hibar HR RP18 end-capped column (150 × 2.1 mm, 3 μm, thermostated at 30 °C, Merck, Darmstadt, Germany) in a LC system that is composed of a solvent degasser (SCM1000, Thermo Scientific, Waltham, MA, USA), a binary high-pressure pump (1100 series, Agilent Technologies, Santa Clara, CA, USA) and a Surveyor autosampler thermostated at 4 °C (Thermo Scientific), and equipped with a UV–visible photodiode array detector (UV6000 LP, Thermo Scientific) and an ion trap mass spectrometer with electrospray ionization source (LCQ Deca, Thermo Scientific). The separation of phenolic compounds was performed using a gradient mixture of A (0.1% v/v formic acid in water) and B (0.1% v/v formic acid in acetonitrile) at a flow rate of 0.2 mL/min. The linear gradient elution steps were as follows: 0–3 min, 3% B; 3–21 min, 7% B; 21–27 min, 13% B; 27–41 min, 20% B; 41–51 min, 45% B; 51–53 min, 90% B; 53–56 min, 90% B, followed by washing and reconditioning of the column. UV–visible detection was performed in the 240–600 nm range. MS spectra were recorded in the full scan mode with negative ionization mode on *m*/*z* 50–2000 range. The source parameters were set as follows: spray voltage, 4.2 kV; capillary voltage, −41 V; sheath gas, 66 arbitrary units; auxiliary gas, 10 arbitrary units; and capillary temperature: 250 °C. The phenolic compounds were identified by comparison of their retention times, UV–vis spectra, and mass spectra with those of the standards, and quantified using the UV–visible spectra based on the external standards for each class of phenolic compounds. Data were analyzed using Xcalibur software (Thermo Scientific).

### 2.6 Analysis of Covalently Bound Phenolic Compounds

The covalently bound phenolic compounds were released by alkaline hydrolysis of HMWF, non-defatted HMWF, and coffee brew according to the method described by^26^ with some modifications. Briefly, 45 mg of sample was dissolved in 3 mL of 2 M NaOH solution containing 20 mM EDTA and 2 w/v % ascorbic acid. After incubation at 30 °C for 1 h, the mixture was adjusted to pH 3.0 with 5 M HCl. The mixture was stored at 4 °C for 2 h, followed by centrifugation at 4000*g* and 4 °C for 10 min. The supernatant was diluted by two with methanol, filtered with a 0.45 μm PTPE filter, and injected (2 μL) into the LC-DAD-MS system for analysis as described in Section 2.5.

### 2.7 Interfacial Activity

The interfacial tension between the stripped rapeseed oil and different coffee fractions in water (0.01 w/v %) was measured with an automated drop volume tensiometer (Tracker, Teclis, Longessaigne, France). A rising oil drop (area: 40 mm^2^ made with a 20-gauge needle) was immersed in an aqueous phase with the component of interest. The interfacial tension (γ) was calculated based on the shape of the droplet using the Laplace equation and measured for 7200 s at 20 °C. The results were expressed as surface pressure (π = γ_0_ – γ), with γ_0_ the interfacial tension between oil and water without any coffee fraction.

### 2.8 Antioxidant Properties

*1,1-Diphenyl-2-picryl-hydrazyl (DPPH) radical scavenging activity* of coffee fractions was determined according to the method described by^27^ with a few modifications. Briefly, 1 mL of fresh DPPH solution (200 μM in ethanol) was added to 1 mL of 0.01 w/v % WPI or coffee fraction suspension/solution in water. The mixture was shaken at 20 °C in the dark for 30 min (Eppendorf ThermoMixer C, Eppendorf, Hamburg, Germany). The absorbance of the reaction mixture (*A*_s_: 1 mL ethanol, 1 mL sample with 0.01 w/v % component) was determined at 517 nm using ethanol as the blank. The scavenged percent of DPPH radicals (%) was calculated according to eq 1.
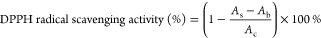
1where *A*_b_ is the absorbance of the mixture of ethanol (1 mL) and sample (1 mL, 0.01 w/v %) and *A*_c_ is the absorbance of the mixture of DPPH solution (1 mL) and water (1 mL).

*Iron chelating capacity* was determined using a modified version of.^28^ In brief, 1 mL of 0.01 w/v % WPI or coffee fraction was mixed with a known amount of ferrous iron solution (1 mL, 5 g/L). The mixture was vortexed and left at 20 °C for 24 h and then separated using an ultrafiltration-centrifugation tube with a membrane (cutoff 10 kDa). The filtrate obtained (0.5 mL) was added to 1 mL of dissociating agent [containing 0.5 mL of 0.5 M l-ascorbic acid and 0.5 mL of 1.4 M acetic acid buffer (pH 4.5)] and 0.1 mL of 6 mM ferene solution. After 5 min, the absorbance was measured at 593 nm. The quantity of bound iron (μg per mg of the sample) was calculated using a mass balance between unbound Fe^2+^ in the filtrate and the initial Fe^2+^ content.

### 2.9 Emulsion Preparation

A coarse O/W emulsion containing 10 wt % stripped rapeseed oil and 90 wt % aqueous phase (with 2 wt % coffee fractions or WPI) was prepared using a rotor-stator homogenizer (Ultra-TURRAX IKA T18 basic, Germany) at 11,000 rpm for 1 min. A M-110Y Microfluidizer (equipped with a F12Y interaction chamber, Microfluidics, Massachusetts, USA) was used to further break down the coarse oil droplets to fine droplets with five passes at 800 bar. Potassium sorbate (0.2 wt %) was added to emulsions to prevent microbial spoilage. Emulsions (2 g aliquots) were partitioned in polypropylene tubes (Eppendorf,15 × 120 mm), which were then incubated in the dark at 40 °C for 7 days under rotative agitation at 2 rpm (SB3 rotator, Stuart, Staffordshire, UK).

*Addition of components.* Stock WPI-stabilized emulsions (with 20 wt % rapeseed oil and 1 wt % WPI in the aqueous phase) were prepared as previously described.^29^ Coffee fractions suspensions, WPI solution, or water were added to the stock emulsions to achieve final concentrations of 0.5 wt % emulsifier, 10 wt % oil, and 0.125–2 w/v % excess compounds (coffee fractions or WPI) in the continuous phase. To prevent microbial growth, 0.2 wt % of potassium sorbate was added. These emulsions were incubated under the same conditions as described above.

### 2.10 Physical Properties of Emulsions

The physical properties of emulsions were measured immediately after emulsification and at the end of incubation at 40 °C.

*The droplet size distribution* was determined by static light scattering using a particle size analyzer (Mastersizer 3000, Malvern Instruments Ltd., Worcestershire, UK). The optical parameters were a dispersed phase refractive index of 1.473, a droplet absorbance of 0.01, and a continuous phase refractive index of 1.33.

*Light microscopy* (Carl Zeiss Axio Scope A1, Oberkochen, Germany) was used to capture the emulsion microstructure. One droplet of the emulsion was placed on a microscopic slide and covered with a coverslip. Images were taken at a magnification of 40×.

*Surface charge* was measured through zeta-potential using a dynamic light scattering instrument (Zetasizer Ultra, Malvern Instruments Ltd., Worcestershire, UK). Emulsions were diluted 1000-fold in water to prevent multiple scattering. The optical parameters were the same as those used for the droplet size distribution measurement. Measurements were performed at 20 °C.

### 2.11 Lipid Oxidation

Lipid oxidation of emulsions was evaluated by determining the primary (lipid hydroperoxides) and secondary (aldehydes) oxidation products throughout the incubation period.

*Lipid hydroperoxides* were measured according to a method reported by^30^ with some modifications. In short, 0.3 g of emulsion was mixed with 1.5 mL of *n*-hexane/2-propanol (3:1, v/v). The mixture was vortexed three times for 10 s each, with 20 s intervals, followed by centrifugation at 14,600 rpm for 2 min. Then, 0.2 mL of the upper organic phase was mixed with a 2.8 mL of methanol/1-butanol (2:1, v/v) and 30 μL of thiocyanate/ferrous iron solution (1:1, v/v). After 20 min, the absorbance of the sample was measured at 510 nm using a DU 720 UV–visible spectrophotometer (Beckman Coulter, Woerden, the Netherlands). The lipid hydroperoxide concentration was calculated using a cumene hydroperoxide standard curve.

*Aldehydes* were measured through the *para*-anisidine value (pAV) according to the AOCS Official Method CD 18–90.^31^ In brief, 2 g of emulsion was mixed with 5 mL of *n*-hexane/2-propanol (3:1, v/v) and 1 mL of saturated sodium chloride solution. The mixture was vortexed three times for 10 s with 20 s intervals and centrifuged at 4000 rpm for 8 min. The absorbance of the upper hexane layer (*A*_*b*_) was measured at 350 nm using hexane as a blank. Then, 1 mL of this hexane phase was mixed with 0.2 mL of *para*-anisidine solution (0.25 w/v % in acetic acid). After 10 min, the absorbance (*A*_*s*_) was measured at 350 nm using hexane with the *para*-anisidine solution as a blank. The pAV (arbitrary units) was calculated according to eq 2
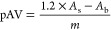
2where *m* is the mass (g) of oil per mL of hexane.

### 2.12 Statistical Analysis

All analyses were carried out in triplicate on at least two independent samples, and data were reported as mean values ± standard deviation. Significance of the results (*p* < 0.05) was determined by one-way analysis of variance (ANOVA) with Tukey’s post hoc test using IBM SPSS statistics software 23.0.0.2 (SPSS Inc, Chicago, Illinois, USA).

## 3 Results and Discussion

### 3.1 Characterization of Coffee Fractions

#### 3.1.1 Chemical Composition

The carbohydrate, protein, and phenolic contents of different coffee fractions are listed in Table 1. The carbohydrate contents of the coffee brew, HMWF, and LMWF were around 38, 70, and 16 wt %, respectively, which are within the range of values reported in literature.^24,32−35^ The majority of the carbohydrates from coffee brew ended up in HMWF (Table 1), indicating that coffee brew carbohydrates are mostly polysaccharides with a minor fraction of simple sugars and oligosaccharides. Mannose, galactose, and arabinose are the most abundant sugar residues in HMWF, which suggests that these are the main constituents of polysaccharides in coffee brew and HMWF.^24,36^ During coffee roasting, these polysaccharides undergo structural changes (e.g., depolymerization, debranching, isomerization, and polymerization) and are involved in melanoidin formation.^37,38^ The most abundant sugars in LMWF were reported to be mannose and galactose.^39^

**Table 1:** Composition of Coffee Fractions^a^

sample	carbohydrates (wt %)	phenolic compounds (wt %)^b^	proteins (wt %)
coffee brew	37.77 ± 0.64^b^	20.31 ± 1.02^b^	17.30 ± 1.25^ab^
HMWF	70.82 ± 3.63^a^	16.94 ± 0.76^bc^	12.18 ± 1.37^b^
non-defatted HMWF	72.85 ± 1.17^a^	15.89 ± 1.08^c^	10.92 ± 0.12^b^
LMWF	16.40 ± 0.50^c^	32.30 ± 1.30^a^	22.29 ± 4.07^a^

a Different letters indicate significant differences (*P* < 0.05) between samples for each component.

b CGA was used as a reference phenolic compound.

The protein contents of the coffee brew, HMWF, and LMWF are 17, 12, and 22 wt %, respectively (Table 1), which is in line with other studies.^24,40^ Proteins in green coffee beans undergo denaturation, depolymerization, and Maillard reactions during roasting, resulting in compositional and structural changes and integration into the polymeric structure of melanoidins.^41^

Coffee brew, HMWF, and LMWF contain ∼20, 17, and 32 wt % of phenolic compounds, respectively (Table 1), which is in line with the findings of.^24^ Potentially, these proportions are overestimated because of interferences with non-polyphenolic materials, in particular proteins, in the Folin–Ciocalteu assay. Therefore, phenolic compounds in their free and bound forms were also analyzed by HPLC-DAD-MS. Free phenolic compounds in coffee brew and LMWF were directly analyzed in aqueous methanol, whereas bound phenolics in HMWF and defatted HMWF were analyzed after alkaline hydrolysis (Table 2). The main phenolic compounds in coffee fractions are CGAs that are derived from esterification of quinic acid and cinnamic acids (including caffeic, ferulic, and *p*-coumaric acids), including caffeoylquinic acids (CQAs), dicaffeoylquinic acids (diCQAs), feruloylquinic acids (FQAs), and *p*-coumaroylquinic acids (*p*CoQAs). As shown in Table 2 (detailed data can be found in Table S1), CQAs were present in much higher amounts than FQAs and diCQAs, which is in agreement with Ludwig et al.^42^ Free CQAs, FQAs, diCQAs, and caffeoylquinolactones (CQLs) were detected in coffee brew and its LMWF, which is consistent with the findings from previous research.^40^ In contrast, no free CGAs were found in HMWFs; during coffee bean roasting, a part of the CGAs is degraded into phenol derivatives keeping their catechol function and bound to melanoidin backbones through covalent linkages (in condensed form and ester linked-form) and, to a lesser extent, via electrostatic interactions,^16,43,44^ forming supramolecular assemblies which cannot pass the ultrafiltration membrane. Thus, covalently bound CGAs were measured after the alkaline hydrolysis of HMWFs coffee fractions. For these fractions, the release of caffeic (CA), ferulic (FA), and *p*-coumaric (*pc*oum) acids were observed (Table 2), suggesting the incorporation of CQAs, diCQAs, FQAs, caffeoylferuloylquinic acids (CFQAs), and *p*-coumaroylquinic acids (*p*CoQAs) into melanoidins.^18^

**Table 2:** Unbound and Covalently Bound Phenolic Compounds of Coffee Fractions (g/100g).^a^

	unbound phenolic compounds		covalently bound phenolic compounds	
	coffee brew	LMWF	HMWF	non-defatted HMWF
total CQAs	2.94 ± 0.14	5.50 ± 0.09	nd	nd
total FQAs	0.23 ± 0.02	0.41 ± 0.03	nd	nd
total diCQAs	0.04 ± 0.00	0.07 ± 0.00	nd	nd
total CQLs	0.42 ± 0.03	0.86 ± 0.07	nd	nd
CA	nd	nd	0.44 ± 0.01	0.43 ± 0.02
FA	nd	Nd	0.10 ± 0.00	0.10 ± 0.00
*Pc*oum	nd	Nd	0.01 ± 0.00	0.01 ± 0.00

a nd: not detected; +/– values correspond to standard deviation (n = 3).

Non-defatted HMWF was prepared to investigate how the defatting step would affect the composition of HMWF and the stability of the emulsions prepared with such fractions. As shown in Table 1, the non-defatted HMWF has a similar amount of carbohydrates, phenolic compounds, and proteins compared to the HMWF. Therefore, we expected that if oil was present, this may not affect the physical properties of emulsions but may negatively affect oxidative stability.

#### 3.1.2 Interfacial Activity

**Figure 1:**
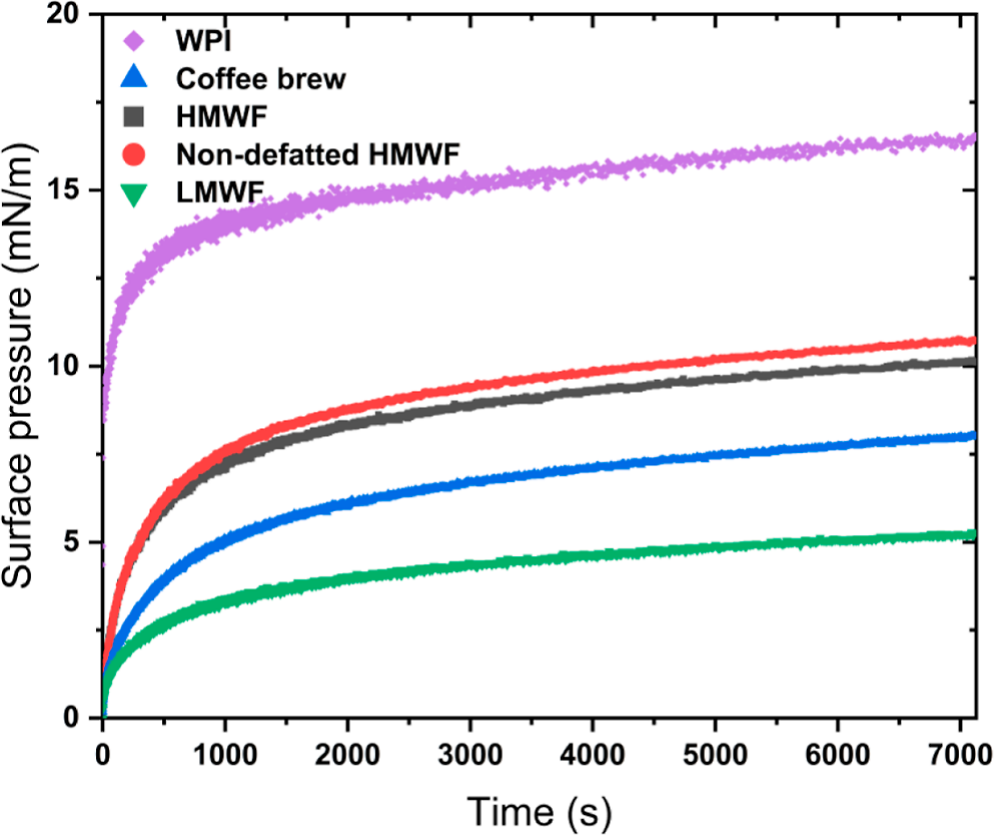
Surface pressure of WPI and coffee fractions (0.01 w/v % in water) as a function of time, at the stripped oil–water interface, at 20 °C. For clarity, one representative curve is shown for each sample, but similar results were obtained on independent triplicates.

The interfacial activity of coffee fractions was determined by their time-dependent capacity to increase the surface pressure at the oil–water interface and was compared with that of WPI, a commonly used emulsifier. As shown in Figure 1, all samples showed a rapid increase in the surface pressure within the first 400 s, followed by a slower increase. However, the surface pressure values obtained with WPI were always higher than those obtained with the coffee fractions. Comparing the coffee fractions, HMWF and non-defatted HMWF led to a higher surface pressure than LMWF, while the surface pressure obtained with coffee brew fell in between those obtained with HMWF and LMWF (Figure 1).

Whey proteins are known to rapidly diffuse and adsorb at the oil–water interface, thus lowering interfacial tension, and in later stages also re-arranging and forming surface films.^45^ HMWF contains amphipathic proteins (e.g., arabinogalactan proteins) as part of the melanoidins and these components are also surface-active.^46^ The majority of compounds in the LMWF are highly polar,^40^ and this may imply that surface activity is relatively low (Figure 1). The lower surface activity of coffee brew compared to HMWF is a logical combination of the effects found for HMWF and LMWF.

### 3.2 Coffee Fractions at the Interface of Emulsions

#### 3.2.1 Physical Properties of Emulsions

HMWF from coffee brew (melanoidins) used at concentrations ranging from 0.25 to 4 wt % was previously found to be able to physically stabilize O/W emulsions, among which the 2 wt % melanoidin-stabilized emulsions showed the highest physical stability.^21^ Here, we tested all coffee fractions at a concentration of 2 wt % for emulsion preparation, and evaluated their effect on droplet size distribution, microstructure, droplet surface charge, and later also lipid oxidation was monitored throughout storage. WPI (2 wt %)-stabilized emulsions were used as reference emulsions.

**Figure 2:**
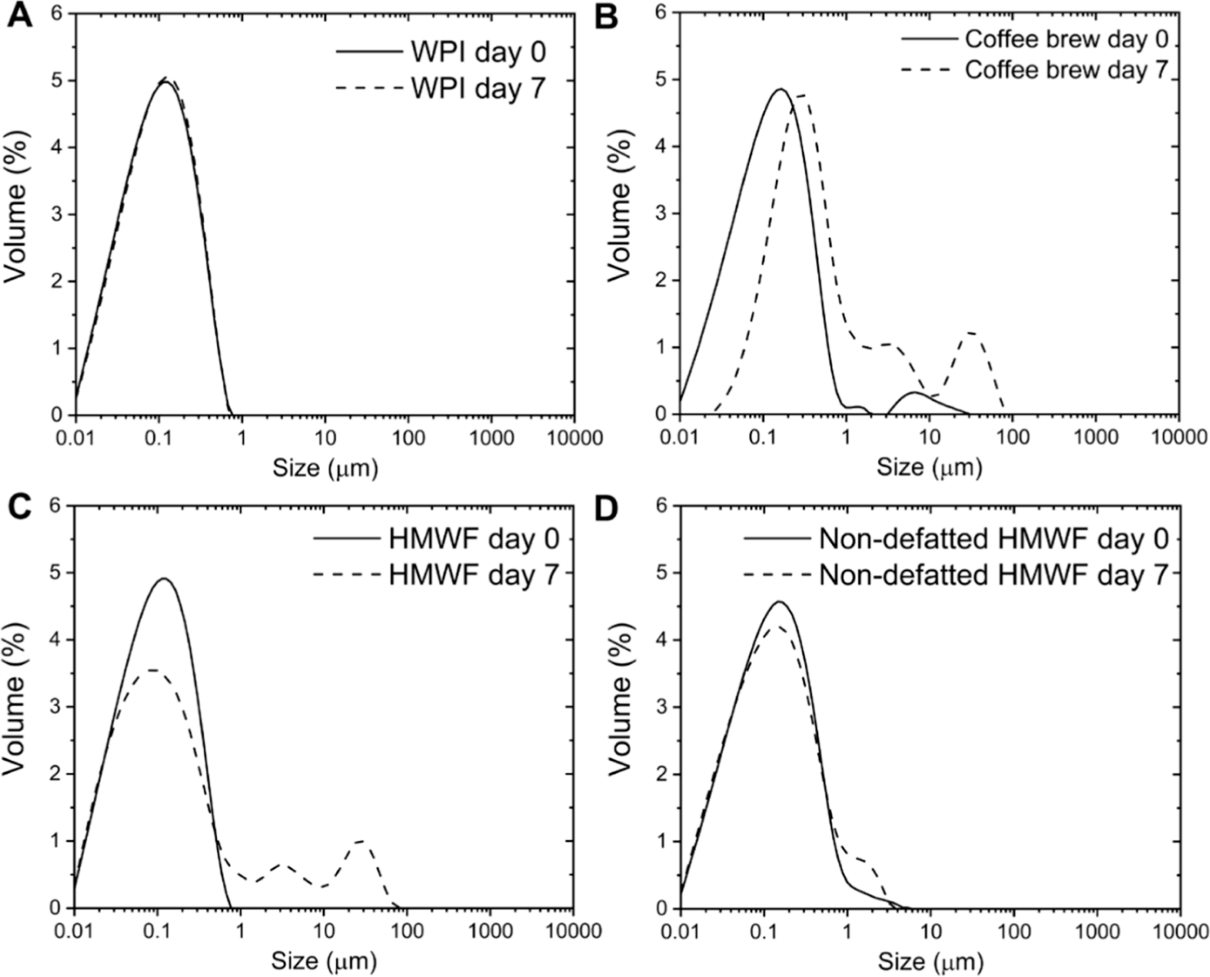
Droplet size distribution of emulsions stabilized with WPI (A), coffee brew (B), HMWF (C), and non-defatted HMWF (D) freshly prepared (solid line) or after 7 days at 40 °C (2) (dotted line). For all emulsions, the concentration of the emulsifying ingredient was 2 wt %. For clarity, one representative curve is shown for each sample, but similar results were obtained on independent triplicates.

**Figure 3:**
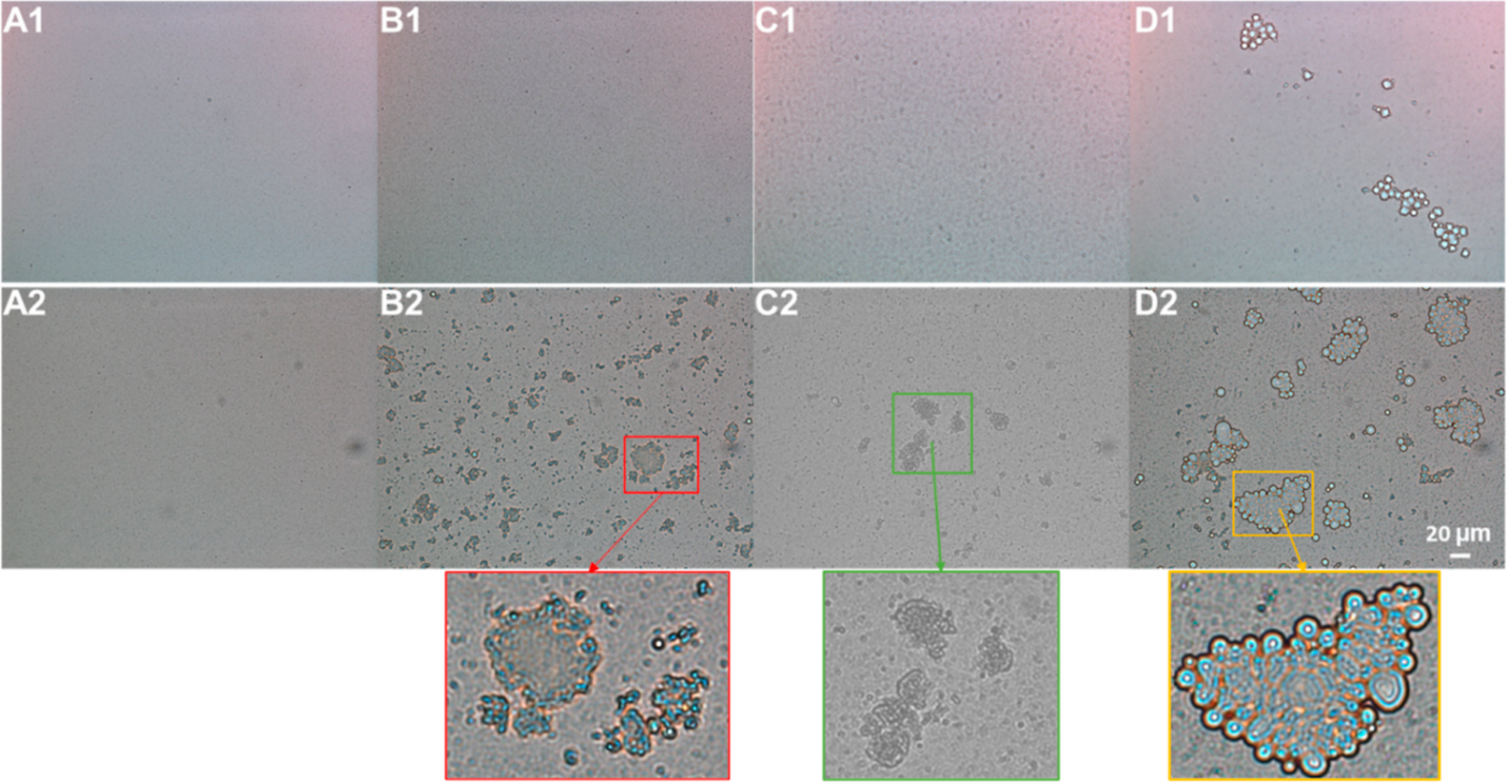
Microscopic pictures of emulsions stabilized with WPI (A), coffee brew (B), HMWF (C), and non-defatted HMWF (D) freshly prepared (1) or after 7 days at 40 °C (2).

With the exception of emulsions stabilized with LMWF that underwent creaming and subsequent oiling off shortly after homogenization, all other freshly prepared emulsions exhibited a nearly monomodal size distribution with a mean droplet size (*d*_*3,2*_) of ∼0.1 μm (Figure 2). The coffee brew-stabilized emulsions showed a small peak at larger sizes due to slight flocculation and coalescence (Figures 2B and 3). Upon 7 days of storage at 40 °C;, WPI-stabilized emulsions remained fully stable (Figure 2A), whereas multimodal size distributions were observed in emulsions stabilized with coffee brew and HMWF (Figure 2B,C), which was probably caused by flocculation and coalescence of droplets (Figure 3). For the non-defatted HMWF-stabilized emulsions, their droplet size distribution profile remained mostly stable even though a minor tail in the size distribution appeared between 1 and 3 μm (Figure 2D). This does not reflect the observation of large structures in optical microscopy, which look like compact flocs or even oil droplets attached onto solid template structures (Figure 3D2). The reason why such structures were not detected in static light scattering may be because their occurrence was still minor enough not to contribute substantially to the signal measured, or they creamed/sedimented so fast that they were not detected. Nevertheless, no oiling off was detected in all emulsions.

**Figure 4:**
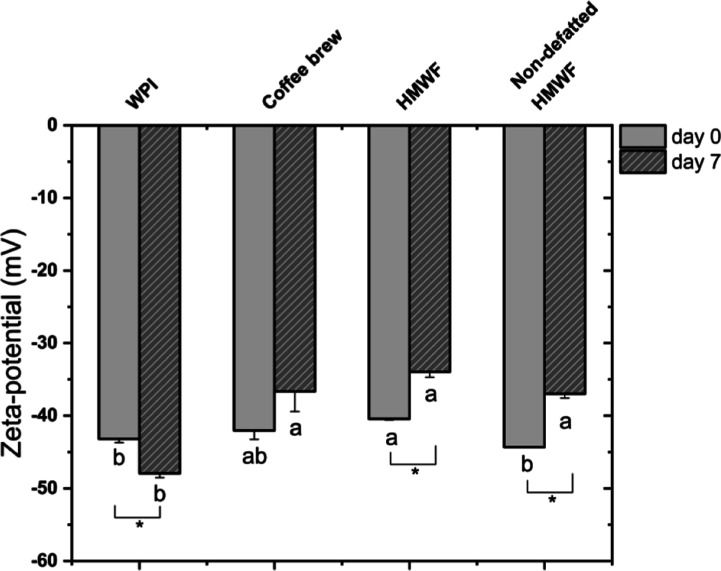
Zeta-potential of the emulsions freshly prepared or after 7 days at 40 °C. The lowercase letter is for comparison among different emulsions. Different letters indicate significant differences (*P* < 0.05). Asterisks indicate a significant difference for the same sample between day 0 and day 7.

All freshly prepared emulsions had a negative zeta-potential around −40 mV (Figure 4), which was expected because both WPI and coffee melanoidins are negatively charged at pH higher than the isoelectric point (∼5.1 and ∼2.5, respectively). At the end of storage, the emulsions still had a considerable net charge, with significant changes noted (except for coffee brew-stabilized emulsions, Figure 4). The decrease in zeta-potential for WPI-stabilized emulsions might be related to the surface-active fatty acids that may be formed upon lipid hydrolysis, or organic acids generated as a result of lipid oxidation, or degradation of positively charged amino groups.^47−49^ In addition, this decrease could also be related to the conformational rearrangements of the whey proteins at the interface, which may lead to an exposure of negatively charged amino groups. The increase in zeta-potential for coffee fraction-stabilized emulsions is most probably the result of a small decrease in pH which reduces the net charge, and this may also favor aggregation of oil droplets (Figures 2 and 3).

#### 3.2.2 Antioxidant Activity of Coffee Fractions

**Figure 5:**
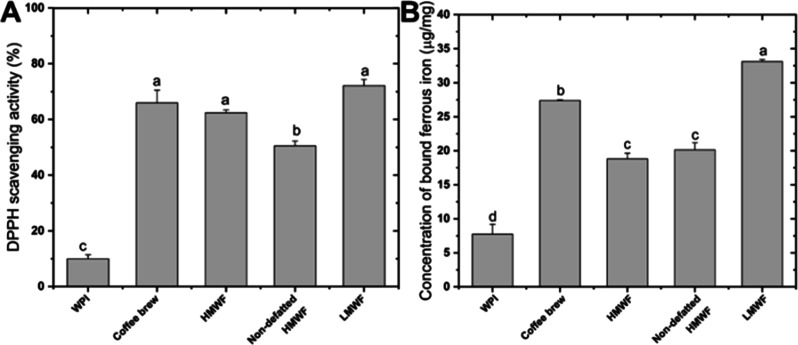
DPPH radical scavenging activity (A) and iron-chelating capacity (B) of WPI and different coffee fractions. The lowercase letter is for comparison among the samples. Different letters indicate significant differences (*P* < 0.05).

Coffee components may affect oxidative reactions through various mechanisms including scavenging of free radicals and binding of metal ions. Therefore, before analyzing the lipid oxidation in emulsions, the antioxidant properties of coffee fractions were assessed and compared with those of WPI. As can be seen in Figure 5A, WPI exhibited a significantly lower DPPH radical scavenging activity than any of the coffee fractions, among which non-defatted HMWF showed the lowest activity. With respect to the iron-chelating activity, all coffee fractions were able to bind more iron than WPI (Figure 5B), with LMWF having a significantly higher capacity than the other fractions (Figure 5B).

For WPI, it has been suggested that the sulfhydryl groups located on the surface of the molecules have hydrogen-donating ability,^50^ whereas the carboxyl groups of acidic amino acids (aspartic acid and glutamic acid) might account for metal-chelating ability.^51^ The antioxidant properties of the HMWF are probably due to the melanoidins that contain CGAs. The presence of reductons, enaminol, and hydroxyl groups in phenolic compounds might explain the strong radical scavenging activity,^52^ whereas the catechol moieties from incorporated phenolic compounds and the ketone and/or hydroxyl groups of pyranone or pyridone might act as metal chelators.^53,54^ LMWF is rich in unbound phenolic compounds, especially those with catechol moieties (e.g., CQAs, Section 3.1) which are effective free radical acceptors and metal chelators.^17,55,56^ In addition, the volatile heterocyclic compounds (e.g., pyrrols, furans, and thiophenes) and hydroxybenzenes (e.g., ethylcathecol and pyrogallol) in LMWF may contribute to over antioxidant activity.^18^

#### 3.2.3 Lipid Oxidation in Emulsions

**Figure 6:**
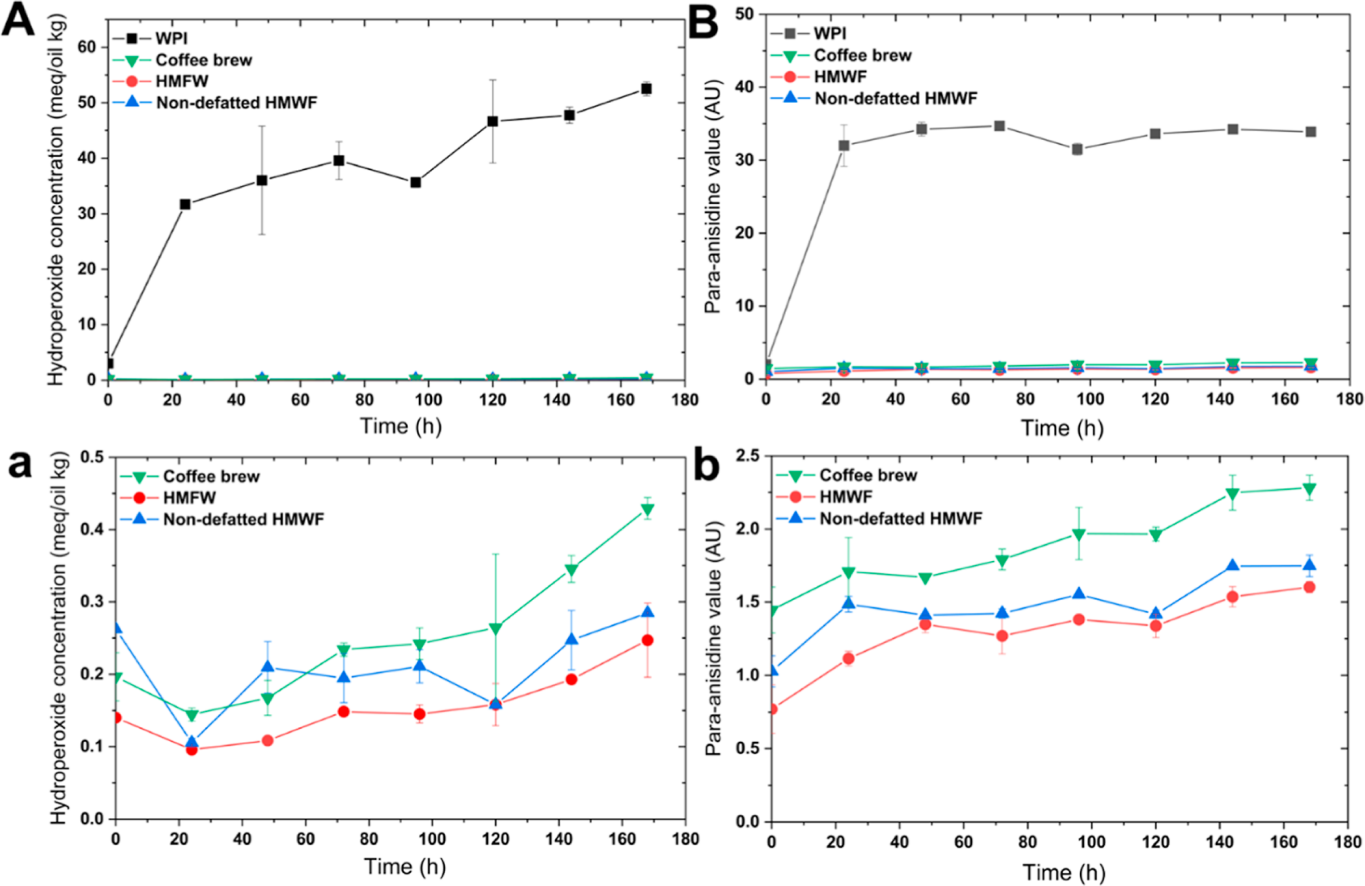
Hydroperoxide concentrations (left column) and *para*-anisidine values (right column) in different emulsions over the incubation period (40 °C, 7 days). Top row: all emulsions; bottom row: coffee fraction-stabilized emulsions (bottom row graphs show a magnification on low values of oxidation markers; please note the difference in Y-axis scales between panels A/a and panels B/b).

Hydroperoxide concentration (Figure 6A) and *para*-anisidine value (pAV) (Figure 6B) were used to characterize lipid oxidation in emulsions. WPI-stabilized emulsions showed a rapid initial increase in hydroperoxides, followed by a gradual increase until the end of storage (Figure 6A). Similarly, the pAV of WPI-stabilized emulsions rapidly increased within 1 day of storage, after which it remained constant for the rest of the storage period (Figure 6B). In contrast, the hydroperoxide concentration and pAV of emulsions stabilized by coffee fractions were very low during the accelerated storage at 40 °C (Figure 6A,B; a magnification is therefore shown in Figure 6a,b), indicating that coffee fractions (coffee brew, HMWF, and non-defatted HMWF) were highly effective in preventing oxidation of emulsified lipids.

The strong ability of coffee fractions to protect lipids from oxidation can be related to their relatively high antioxidant activity (compared to WPI, Section 3.2.2) and their interfacial localization. In the early stages of incubation, it is likely that trace amounts of pre-existing lipid hydroperoxides (LOOH) located at the oil–water interface would decompose into alkoxy radicals (LO^•^) or peroxyl radicals (LOO^•^).^57^ Some compounds from the adsorbed coffee fractions (e.g., phenolic compounds and melanoidins, as discussed in Section 3.2.2) might act as chain-breaking electron donors, which could readily transfer hydrogen atoms to scavenge LO^•^ and LOO^•^, thereby inhibiting lipid oxidation.^58,59^ On the other hand, coffee fractions have metal binding capacity (Figure 5B), which prevents metals from initiating radical formation and decomposing surface-active LOOH. Both relatively high radical scavenging and iron binding capacities seem to be logical explanations for the effectiveness of coffee fractions, whereas the much lower values for WPI are consistent with the greater oxidizability of the corresponding emulsions.

Next to the role of the adsorbed fractions, components present in the continuous phase may also play an important role in lipid oxidation.^20^ For example, melanoidins may trap transition metals and free radicals in the continuous phase and thus prevent these aqueous pro-oxidants from getting into contact with labile unsaturated lipids in the droplets. To distinguish between these effects, excess coffee material was added to the continuous phase of preliminary prepared emulsions, and both physical and oxidative stability were monitored.

### 3.3 Added Coffee Materials to the Continuous Phase of Emulsions

#### 3.3.1 Influence of HMWF Concentrations on the Stability of Emulsions

Stock WPI-stabilized emulsions were prepared with minimal amounts of unadsorbed WPI remaining in the continuous phase,^29^ and HMWF suspensions with concentrations ranging from 0 to 2 w/v % were added to the emulsion after homogenization. Physical stability and lipid oxidation were monitored during storage at 40 °C for 4 days.

**Figure 7:**
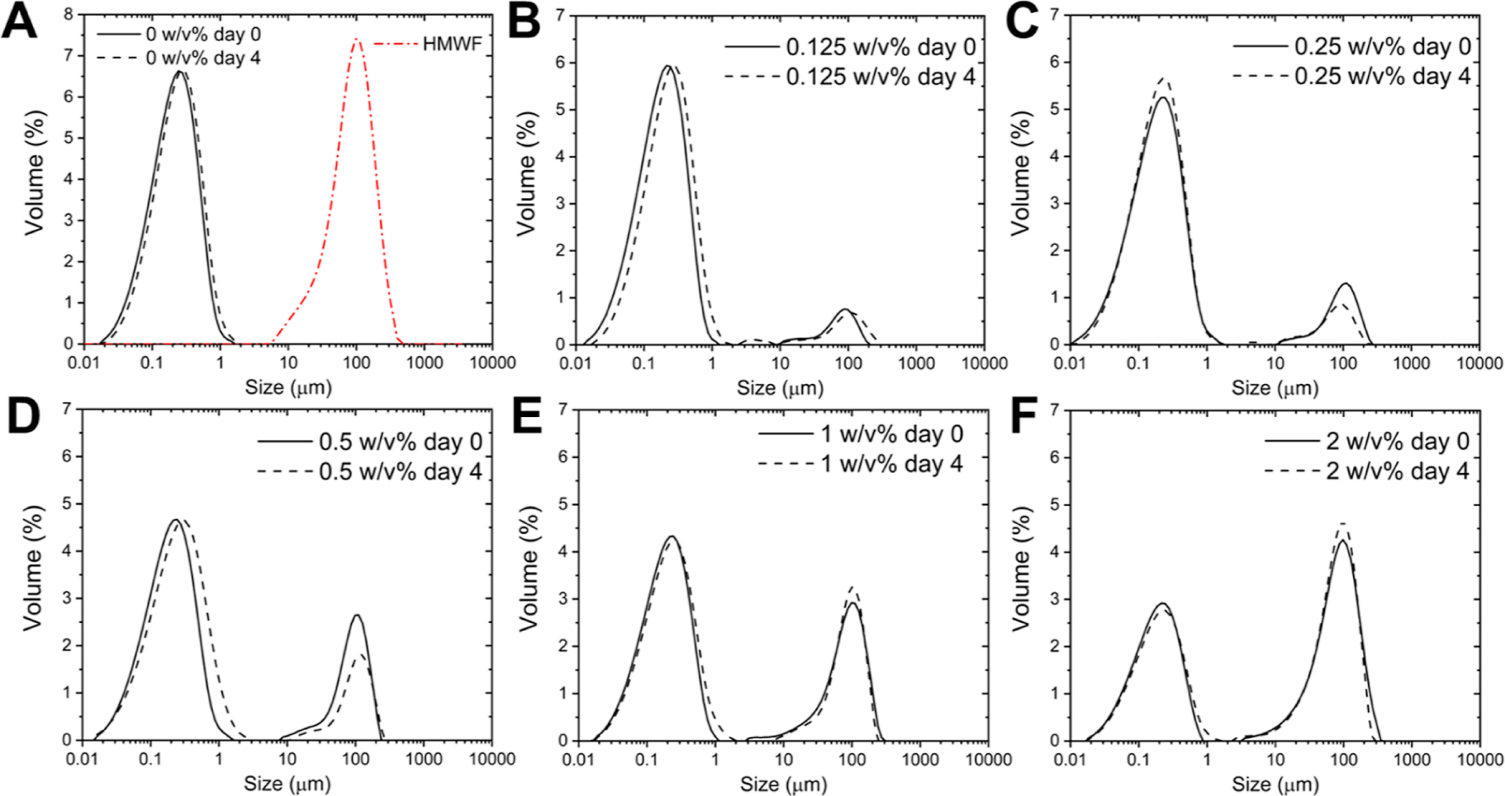
Droplet size distribution of WPI-stabilized emulsions stabilized with 0 (A), 0.125 (B), 0.25 (C), 0.5 (D), 1 (E), and 2 (F) w/v % HMW coffee melanoidins added to the emulsion post-homogenization. For clarity, one representative curve is shown for each sample, but similar results were obtained on independent triplicates. The red curve in (A) is corresponding to the particle size distribution of the HMWF dispersion.

All emulsions with added HMWF exhibited bimodal size distributions (Figure 7): the peak ranging from 0.01 to 1 μm corresponds to the emulsion droplets, and the second peak to aggregated HMWF in the continuous phase. This is supported by (i) the similar particle size distribution of the HMWF dispersion (Figure 7A, red curve) and (ii) the increased intensity of the second peak as HMWF concentration increased (Figure 7). No appreciable changes in droplet size and microstructure were observed for all emulsions upon storage (Figures 7 and S1), suggesting these emulsions were physically stable.

**Figure 8:**
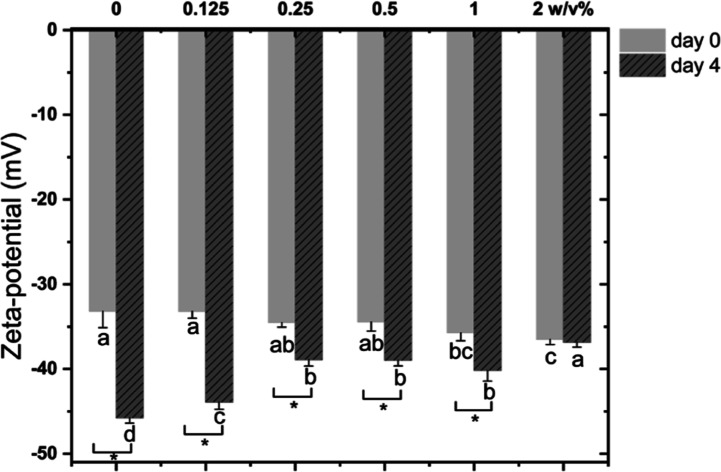
Zeta-potential of the WPI-stabilized emulsions supplemented with 0–2 w/v % of HMWF freshly prepared or at the end of the incubation period (40 °C, 4 days).

As can be seen in Figure 8, emulsion droplets had a slightly more negative surface charge when the HMWF concentration increased, which may be due to some exchange taking place at the interface, leading to more negatively charged “compounds” from HMWF, such as uronic acids from arabinogalactans and ferulic acid or caffeic acid moieties from CGAs incorporated at the interface.^43^ The decrease in zeta-potential over time (Figure 8) can be similarly explained as before by the formation of fatty acids or organic acids, or degradation of the positively charged amino groups, or the conformational rearrangements of the whey proteins (Section 3.2.1).

**Figure 9:**
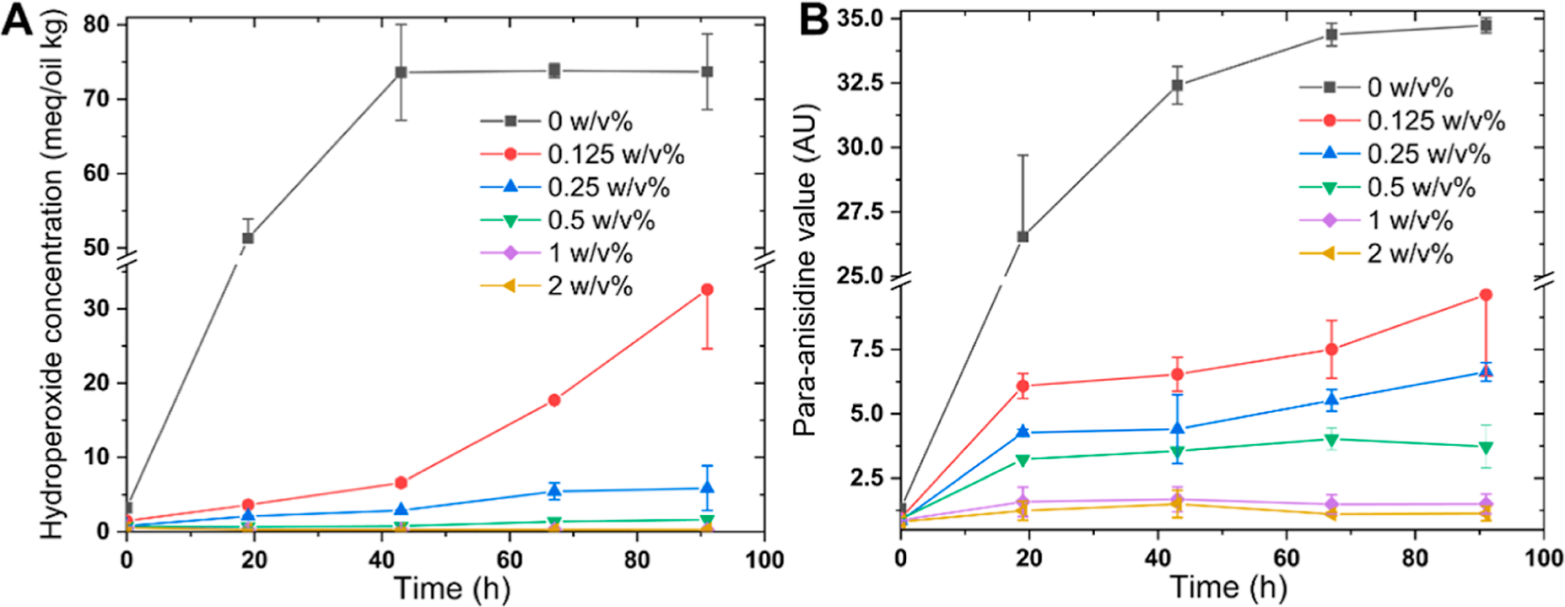
Hydroperoxide concentrations (A) and *para*-anisidine values (B) in WPI-stabilized emulsions supplemented with 0 to 2 w/v % of HMWF, over the incubation period (40 °C, 4 days).

With respect to lipid oxidation, hydroperoxides and aldehydes developed earlier, faster and to a much greater extent in the control emulsion (0 w/v % HMWF) than in the other emulsions (supplemented with 0.125–2 w/v % HMWF) (Figure 9), in which oxidation products were formed according to the amount of HMWF added. It is actually challenging to compare the effects since the difference between the curves is highly time-dependent (as would be expected for cascaded reactions like lipid oxidation). When taking the final concentrations measured, the aldehyde contents were 3.6-, 5.2-, 9.3-, 23-, and 31-fold higher for the control emulsion than emulsions to which HMWF was added at 0.125, 0.25, 0.5, 1, and 2 w/v %, respectively. What is more important to point out is that HMWF was highly efficient even at very low concentrations used in the continuous phase. Most probably, the free radical scavenging and iron-binding abilities (Figure 5A,B) are instrumental in creating such positive effects, as also discussed before.^60^

#### 3.3.2 Influence of Coffee Fractions on the Stability of Emulsions

In the last experiment, the different coffee fractions were added to whey protein-stabilized emulsions, post-homogenization, to test their capacity to inhibit lipid oxidation at a concentration of 0.25 w/v %. Again, no appreciable differences in the physical properties (droplet size distribution, microstructures, and zeta-potentials) were observed during incubation for all emulsions (Figures S2–S4).

**Figure 10:**
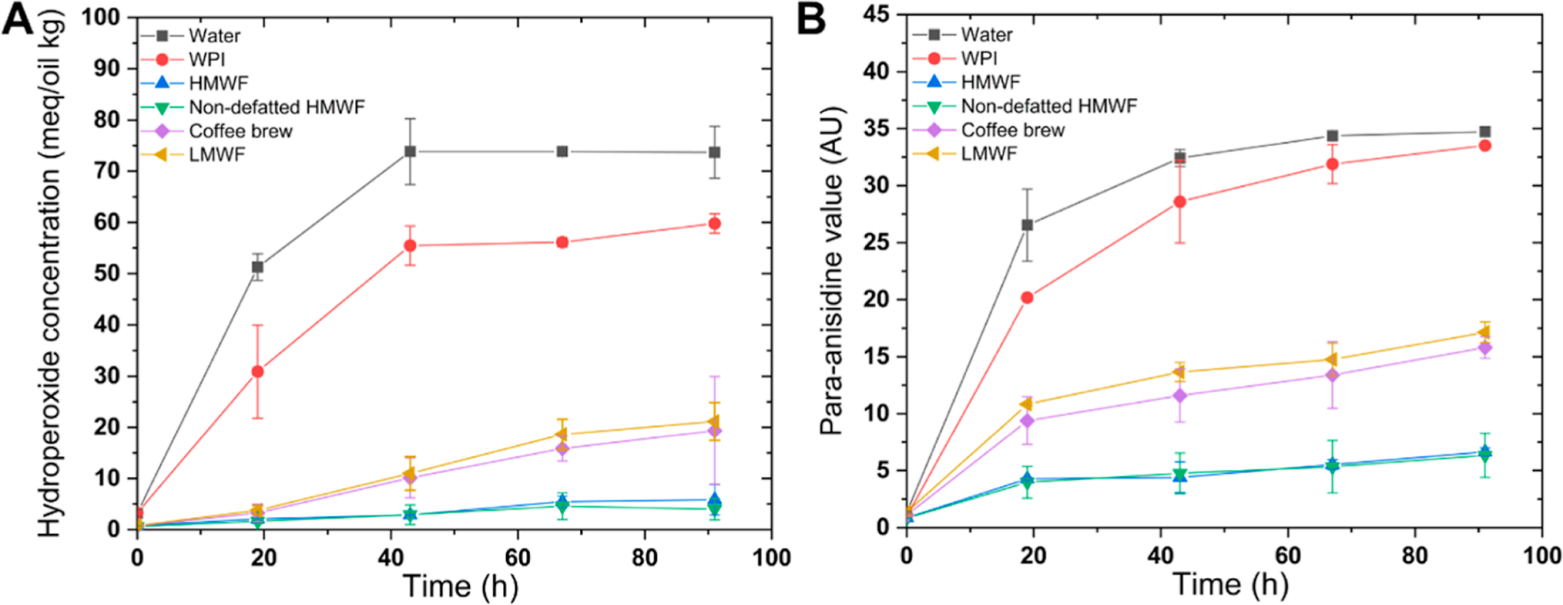
Hydroperoxide concentrations (A) and *para*-anisidine values (B) in WPI-stabilized emulsions supplemented with excess WPI or various coffee fractions (0.25 wt %) over the incubation period (40 °C, 4 days).

With respect to lipid oxidation (Figure 10), the presence of added compounds improved the oxidative stability of emulsions, although WPI was considerably less effective than coffee brew and LMWF, which themselves were less effective than both HMWFs. After 4 days of storage, the aldehyde concentration was 1.0-, 2.0-, 2.2-, 5.2-, and 5.5-fold higher for the control emulsion than for emulsions containing excess WPI, LMWF, coffee brew, HMWF, and non-defatted HMWF, respectively (Figure 10B). The order of appearance is not in line with radical scavenging and iron-chelating activities (Figure 5): although LMWF achieves the best antioxidant activities in single phase test systems, it clearly does not in emulsions. This can be due to the partitioning and physical location of the involved components in emulsions systems, which is why such antioxidant tests are often criticized for their (ir)relevance to model or real food systems.

We expect that the positioning of HMWF components at the oil–water interface puts them where their action is needed. Overall, HMWF components may be more likely to bind to the interface than their low-molecular-weight counterparts,^61^ as seems to be confirmed by the surface pressure measurements (Figure 1); in addition, assuming they may locate at the interface, adsorbed HMWFs would be less mobile and thus could be more efficient to prevent lipid oxidation by conferring their antioxidant moieties a more substantial residence time at the interface, as compared to low-molecular-weight molecules.^62^ Besides, as discussed in Section 3.1.1, unbound phenolic compounds were recovered in LMWF, and covalently bound phenolic compounds were found in HMWF. Unbound phenolic compounds themselves might be oxidized by oxygen and transition metals during the storage at 40 °C, whereas bound phenolic compounds may be protected against oxygen by the large moieties (e.g., melanoidin backbones) they are bound to.^63^ In addition, as compared to HMWF, LMWF and coffee brew had higher phenolic contents (Tables 1 and 2), which may result in a higher amount of phenolic compound-bound Fe^3+^^64^ and higher reducing power that reduces Fe^3+^ to Fe^2+^ in emulsions,^49,65^ thereby promoting the formation of free radicals and the decomposition of hydroperoxides. Furthermore, the total antioxidant effect of coffee fractions is due to hydrophilic as well as hydrophobic compounds,^66^ and thus, depending on the polarity of the media, different compounds might be responsible for the tested antioxidant effect. This implies that antioxidants having a high response in the iron-chelating or DPPH assay may have a low response in the emulsion systems due to partitioning effects. Moreover, obviously, there could still be numerous other components at work, resulting in synergism or antagonism of antioxidants.^67^ In spite of this, our finds clearly point to the great potential of coffee fractions to control oxidation in emulsion, either as a main emulsifier, or as an add-on to the emulsion after its preparation.

This work demonstrates the great potential of various coffee fractions in the preparation of emulsions that are physically as well as oxidatively stable. We explored using various fractions as emulsifiers, and as add-ons post emulsification. Especially, the HMWF is able to form emulsions with a nearly monomodal size distribution and keep emulsions chemically stable at 40 °C for 7 days. When added to dairy protein-stabilized emulsions post homogenization, all coffee fractions were able to slow down lipid oxidation considerably without significantly affecting the physical stability of emulsions, though both HMWFs were more effective in retarding lipid oxidation than coffee brew and LMWF.

It is expected that a number of effects, such as the antioxidant properties (metal chelating and radical scavenging activities) of coffee fractions, the partitioning of antioxidant components in the emulsions and the phenolic compounds profile, are responsible for the overall effects that we found. Fractionation into different coffee fractions improves the techno-functional properties of coffee. This research showed that coffee ingredients could act as multifunctional stabilizers with a wide potential for application in dispersed systems (e.g., emulsions) as used in foods, pharmaceutics, and cosmetics. Future work is directed toward understanding the contribution of individual components to the antioxidant activity of coffee fractions.

## Supporting Information Available

Detailed unbound and covalently bound phenolic compounds of coffee fractions; microscopic pictures of WPI-stabilized emulsions with HMWF added to the continuous phase; droplet size distribution of WPI-stabilized emulsions with different fractions added to the continuous phase; microscopic pictures of WPI-stabilized emulsions with different fractions added to the continuous phase; and zeta-potential of WPI-stabilized emulsions with different fractions added to the continuous phase (PDF)
