# Supplementary material for: Physical and Oxidative Stabilization of Oil-In-Water Emulsions by Roasted Coffee Fractions: Interface- and Continuous Phase-Related Effects
Source: J Agric Food Chem. 2023 Mar 9;71(11):4717–28. doi: 10.1021/acs.jafc.2c07365 (PMC10037332; doi:10.1021/acs.jafc.2c07365)
Supplement: Supplementary file 1 — jf2c07365_si_001.pdf [file jf2c07365_si_001.pdf]

[Supporting information]

Physical and oxidative stabilization of oil-in-water emulsions by roasted coffee fractions: Interface- and continuous phase-related effects

Jilu Feng[1, 2], Karin Schroën[2], Sylvain Guyot[4], Agnès Gacel[4], Vincenzo Fogliano[1], Claire C. Berton-Carabin[2, 3]*

[1]Food Quality and Design Group, Wageningen University and Research, Wageningen 6708WG, Netherlands

[2]Food Process and Engineering Group, Wageningen University and Research, Wageningen 6708WG, Netherlands

[3]INRAE, UR BIA, F-44316, Nantes, France

[4]INRA UR1268 BIA, F-35653, Le Rheu, France

*To whom correspondence should be addressed:

Food Process and Engineering Group, Bornse Weilanden 9, 6708 WG, Wageningen, the Netherlands.

E-mail address: claire.berton-carabin@inrae.fr

Table S1. Detailed unbound and covalently bound phenolic compounds of coffee fractions (g/100g).

RT (min)	λ_{max} (nm)	$[M-H]^-$	Compounds	Unbound phenolic compounds		Covalently bound phenolic compounds	
				**Coffee brew**	**LMWF**	**HMWF**	**Non-defatted HMWF**
11.06	320	353	**CQA isomer**	0.11 ± 0.01	0.21 ± 0.00	nd	nd
12.98	320	353	**CQA isomer**	0.62 ± 0.04	1.22 ± 0.00	nd	nd
16.83	320	353	**CQA isomer**	0.23 ± 0.02	0.39 ± 0.02	nd	nd
17.40	320	353	**CQA isomer**	0.09 ± 0.01	0.18 ± 0.00	nd	nd
19.18	320	353	**5CQA**	0.99 ± 0.04	2.06 ± 0.07	nd	nd
19.80	326	367	**FQA isomer**	0.08 ± 0.01	0.16 ± 0.03	nd	nd
20.15	320	353	**CQA isomer**	0.69 ± 0.03	1.44 ± 0.00	nd	nd
22.50	320	179	**CA**	nd	nd	0.44 ± 0.01	0.43 ± 0.02
22.83	320	335	**CQL isomer**	0.03 ± 0.01	0.06 ± 0.00	nd	nd
23.03	320	353	**CQA isomer**	0.03 ± 0.00	nd	nd	nd
28.85	320	353	**CQA isomer**	0.19 ± 0.01	nd	nd	nd
29.60	326	367	**FQA isomer**	0.14 ± 0.01	0.26 ± 0.00	nd	nd
30.70	320	335	**CQL isomer**	0.04 ± 0.00	0.08 ± 0.01	nd	nd
31.13	320	335	**CQL isomer**	0.24 ± 0.02	0.45 ± 0.04	nd	nd
31.60	310	163	***p*coum**	nd	nd	0.01 ± 0.00	0.01 ± 0.00
33.71	320	335	**CQL isomer**	0.11 ± 0.01	0.27 ± 0.03	nd	nd
37.30	320	193	**FA**	nd	nd	0.10 ± 0.00	0.10 ± 0.00
46.51	320	515	**diCQA**	0.04 ± 0.00	0.07 ± 0.00	nd	nd

RT: retention time; nd: not detected; +/- values correspond to standard deviation (n=3).

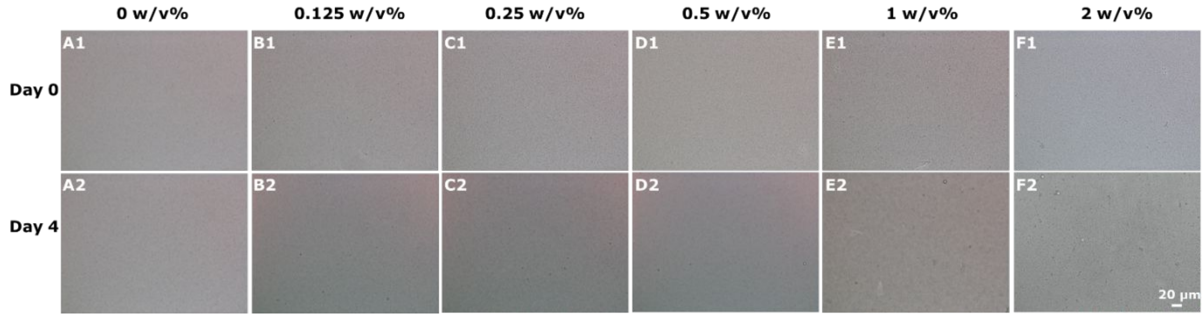

Figure S1. Microscopic pictures of WPI-stabilized emulsions with 0 (A), 0.125 (B), 0.25 (C), 0.5 (D), 1 (E), and 2 (F) w/v% of HMWF added to the continuous phase freshly prepared (1) or at the end (2) of the incubation period (40 °C, 4 days).

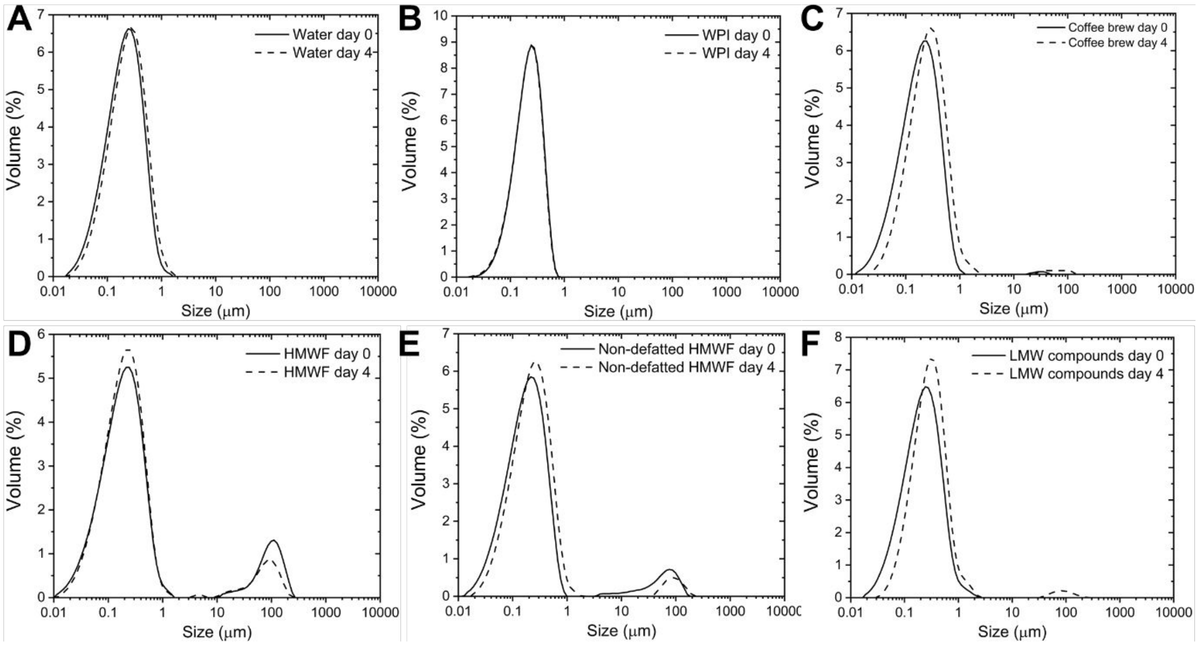

Figure S2. Droplet size distribution of WPI-stabilized emulsions with water (A), WPI (B), coffee brew (C), HMWF (D), non-defatted HMWF (E), and LMWF (F) added to the continuous phase post-homogenization (0.25 wt%). For clarity, one representative curve is shown for each sample, but similar results were obtained on independent triplicates.

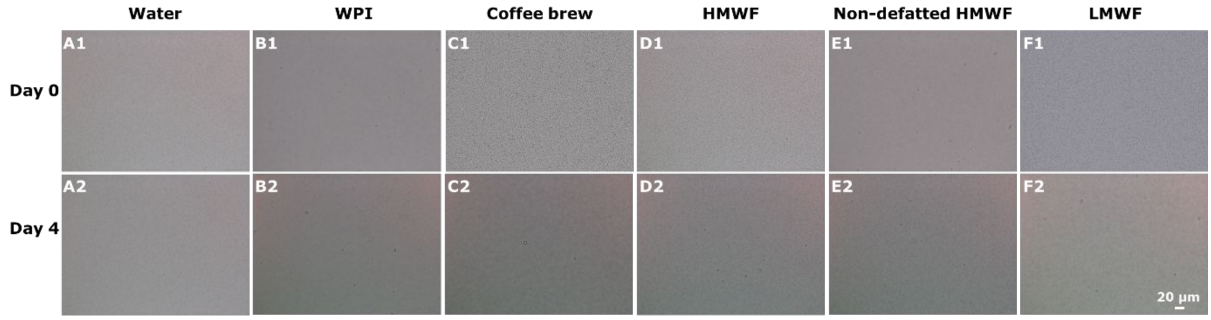

Figure S3. Microscopic pictures of WPI-stabilized emulsions with water (A), WPI (B), coffee brew (C), HMWF (D), Non-defatted HMWF (E), and LMWF (F) added to the continuous phase (0.25 wt%), freshly prepared (1) or at the end (2) of the incubation period (40 °C, 4 days).

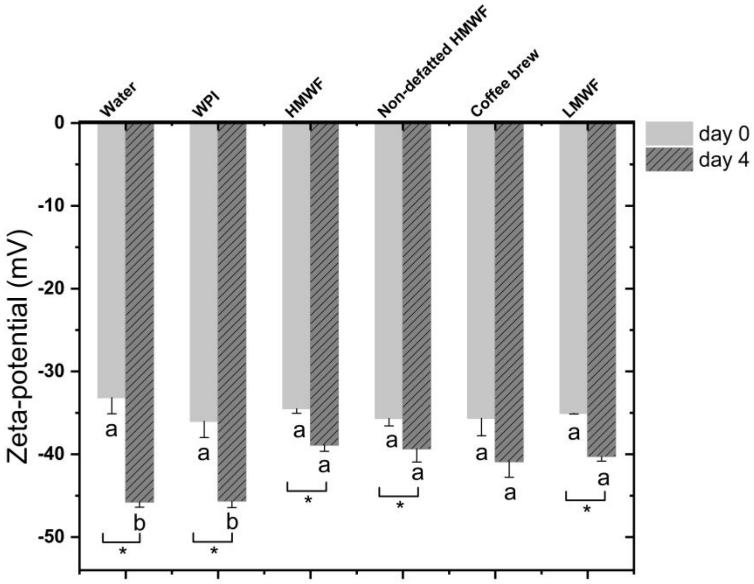

Figure S4. Zeta-potential of WPI-stabilized emulsions supplemented with excess WPI or various coffee fractions (0.25 wt%), freshly prepared or at the end of the incubation period (40 °C, 4 days).
